# Blood‐Based Biomarkers in the Lecanemab Patient Pathway for Early Alzheimer’s Disease in the United States

**DOI:** 10.1002/alz70861_108607

**Published:** 2025-12-23

**Authors:** Cara Leahy, Michael Henry Rosenbloom, Marwan N. Sabbagh, Jose Soria‐Lopez, Gregory Cooper, Samuel Giles, Martin Sadowski, Curtis Schreiber, Paul E Schulz, David C Weisman, Christian J Camargo, Brooke Allen, Courtney Adams, Daryl Jones

**Affiliations:** ^1^ Memorial Healthcare Institute for Neuroscience, Owosso, MI USA; ^2^ University of Washington Memory and Brain Wellness Center, Seattle, WA USA; ^3^ Barrow Neurological Institute, Phoenix, AZ USA; ^4^ University of California San Diego, La Jolla, CA USA; ^5^ The Neuron Clinic, San Diego, CA USA; ^6^ Norton Neuroscience Institute, Louisville, KY USA; ^7^ Memory Treatment Centers, Jacksonville Beach, FL USA; ^8^ New York University Langone Health, New York, NY USA; ^9^ Missouri Memory Center, Citizens Memorial Hospital, Bolivar, MO USA; ^10^ John P. and Kathrine G. McGovern Medical School at UTHealth, Houston, TX USA; ^11^ Abington Neurologic Associates, Abington, PA USA; ^12^ University of Miami Miller School of Medicine, Miami, FL USA; ^13^ Roaring Fork Neurology, Basalt, CO USA; ^14^ Eisai Inc, Nutley, NJ USA; ^15^ Eisai Inc., Nutley, NJ USA

## Abstract

**Background:**

Lecanemab‐irmb (LEQEMBI®) is indicated for the treatment of Alzheimer's disease (AD) in patients with mild cognitive impairment or mild AD, with confirmed amyloid‐beta (Aβ) pathology. Traditionally, Aβ pathology has been confirmed through positron emission tomography (PET) or cerebrospinal fluid (CSF) analysis. More recently, blood‐based biomarkers (BBMs) have emerged as promising diagnostic tools with the potential to facilitate earlier diagnosis of AD. This analysis investigated current real‐world utilization of BBMs in the lecanemab patient pathway in the United States.

**Method:**

This multicenter, retrospective case series and patient pathway study was conducted in 15 geographically diverse neurology clinics, each abstracting deidentified medical chart data for up to 25 patients receiving lecanemab (≥7 infusions) and 1 neurologist per site completing an electronic survey plus an interview. BBM utilization was assessed using deidentified case report forms (CRFs), a survey, and interviews. The survey examined the context of BBM use, including their role in confirming amyloid pathology without further follow‐up or for triage/screening prior to CSF or PET diagnostic testing. This interim analysis (cutoff date: April 11, 2025) includes ∼25% of total expected cases (final cut: May 23, 2025). The protocol received central institutional review board exemption.

**Result:**

In an interim analysis of 94 CRFs, BBMs supported an AD diagnosis in 30% of cases. BBMs were used as a screening/triage tool with follow‐up CSF/PET testing in 27% of cases, and independently to confirm amyloid pathology in 3% of cases. CSF (33%) and amyloid PET (36%) were the primary modalities for confirming AD diagnosis. A survey of 7 respondents showed 4 centers use BBMs for triage and 2 for confirmatory diagnosis. Six centers measure the Aβ42/40 ratio with BBMs, and 3 also analyze *p* ‐tau217 or the *p* ‐tau217 ratio.

**Conclusion:**

These data highlight the evolving implementation of BBMs in clinical practice, with 30% of patients in the interim sample having a BBM help inform a neurologist's diagnosis and decision‐making. In the full data set analysis (data cutoff: May 23, 2025), quantitative data from additional completed surveys and a qualitative analysis from interviews with neurologists on BBM roles in AD diagnosis, testing logistics, insurance coverage, and implementation challenges will be reviewed.

